# Adult-Onset Myoclonus in a Large Urban Inpatient Setting: A Retrospective Cohort Study

**DOI:** 10.5334/tohm.977

**Published:** 2025-01-10

**Authors:** Karin Oh, Moath Hamed, Donna Zarandi, Moyosore Oluleye, Anas Zaher, Jude Elsayegh, Shaheen-Ahmed Rizly, Xiaoyue Ma, Hwai Yin Ooi, Harini Sarva, Miran Salgado, Daryl Victor

**Affiliations:** 1 Department of Neurology, Columbia University Irving Medical Center, New York, NY, US; 2 Department of Neurology, New York Presbyterian Brooklyn Methodist Hospital, Brooklyn, NY, US; 3 Department of Neurology, Weill Cornell Medicine, New York, NY, US; 4 Department of Internal Medicine, New York Presbyterian Brooklyn Methodist Hospital, Brooklyn, NY, US; 5 Department of Population Health Sciences, Weill Cornell Medicine, New York, NY, US

**Keywords:** myoclonus, asterixis

## Abstract

**Background:**

Myoclonus is a hyperkinetic movement with various attributable etiologies, semiologies, and treatment outcomes. To our knowledge, few studies investigated adult-onset myoclonus in an inpatient setting.

**Methods:**

We retrospectively reviewed charts of adult inpatients with myoclonus at New York Presbyterian Brooklyn Methodist Hospital between 2011 and 2021. Data was analyzed with descriptive statistical methods to elucidate etiology-specific demographics and outcomes.

**Results:**

279 individuals, 56.63% female, were included in our study, aging at 70.61 + 15.76 years. More than 50% were not initially diagnosed with myoclonus by the admitting medical team, and more than 50% had 2 or more ascribable etiologies. Symptomatic myoclonus – mostly of toxic-metabolic or hypoxic-ischemic etiology – accounted for most cases. Hypoxic-ischemic etiologies had shorter durations prior to presentation and were also most resistant to treatment. Renal-associated myoclonus was most associated with asterixis, whereas stimulus-sensitive myoclonus was strongly associated with hypoxic-ischemic etiology. Mortality in-hospital was strongly associated with hypoxic-ischemic etiology and least associated with neurodegenerative and idiopathic etiologies. Treatment response rate diminished in patients who were tried on a second or third anti-seizure drug compared to those trialed on one.

**Discussion:**

Myoclonus remains an underdiagnosed hyperkinetic movement disorder with various ascribable etiologies of varying demographic characteristics and treatment outcomes.

Myoclonus is a hyperkinetic movement disorder consisting of brief, shock-like involuntary movements due to muscular contraction(s) or inhibition(s), or so-called “positive” and “negative” (asterixis) myoclonus [1]. It is further subdivided into reflexive (in response to an external or internal stimulus) or spontaneous (independent of external or internal stimulus) subtypes. Myoclonus is also one of the rare movement disorders for which a degree of localization can be established by careful neurological history and examination [2]. Possible localizations include, in descending order of reported prevalence, cortical, psychogenic, subcortical or brainstem reflex, spinal, peripheral, and propriospinal myoclonus [3]. Etiologies are classified as essential (idiopathic, genetic, primary), physiologic (usually an exaggerated phenomenon such as hypnagogic myoclonus), epileptic, or symptomatic (due to an underlying vascular, neurodegenerative, or neoplastic lesion, or systemic toxic, metabolic, or infectious disease) [1,2,3]. Further etiological classification can be aided through history-taking, comprehensive medication review, detailed neurological examination, routine laboratory testing of serum, urine, and/or cerebrospinal fluid, dedicated neuroaxis imaging, and electrophysiological testing [4]. Treatment of myoclonus may be etiology-specific but can also encompass sedatives and anti-epileptic medications [5].

Examination for myoclonus heavily relies on astute clinical observation. The differential diagnoses for myoclonus include other hyperkinetic movement disorders such as chorea, tics, stereotypies, dystonia, and tremor [4]. The brevity and rapidity of myoclonic movements distinguish this near-instantaneous movement disorder from most hyperkinetic movement disorders. Prolonged observation may be necessary to identify involved body parts (axial, appendicular, proximal, or distal), and classify myoclonus based on location (focal, multifocal, segmental, or generalized), synchronicity of movements (whether body parts involved twitch at identical or separate intervals), and triggers of myoclonus (spontaneity, reflex, action or asterixis). Distinguishing features of myoclonus and other hyperkinetic movement disorders are described in Table 1 [6,7,8].

**Table 1:** Distinguishing features of myoclonus and other movement disorders.


	MYOCLONUS	TICS	DYSTONIA	TREMOR	CHOREA

Duration/Cadence of movements	Very brief, shock-like	Brief	Sustained, longer duration	Sustained, longer duration	Could be brief

Onset	Abrupt	Brief	Gradual	Gradual	Rapid

Reflex/Trigger	Frequent	Premonitory urge	May be kinesigenic	Rest, postural, or action	No

Termination	Abrupt	Abrupt	Progressive	Progressive	Progressive

Suppressibility	No	Temporary	No	Temporary	No

Pattern	Simple	Simple/complex	Multiplanar complex	Sinusoidal, may be rhythmic	Flows from one body part to another

Neurophysiological testing	Back-averaging EEG potentials preceding EMG	Organization of the movement	May be useful for treatment	EMG/NCS can distinguish action tremor from myoclonus	Unhelpful


Although well-described in the literature, myoclonus is often misdiagnosed or mischaracterized, with significant inter-rater variability in localization, let alone diagnosis, even amongst movement disorder specialists [9,10,11]. One study of 10 individuals with post-anoxic myoclonus found poor inter-rater agreement amongst physicians attempting to characterize the phenotype and presence of stimulus sensitivity, but substantial agreement for the Unified Myoclonus Rating Scale scores of these individuals [10]. This level of agreement can be enhanced with electrophysiological studies, with another study of 66 individuals using electrophysiologic criteria finding good inter-rater agreement with identifying myoclonus (91%) but poor to moderate inter-rater agreement with localizing or subtyping myoclonus [11].

Various studies have attempted to classify myoclonus from an epidemiological perspective in both outpatient and inpatient settings [3]. However, most studies have classified myoclonus as a subset or part of a larger epidemiological study of movement disorders in general, as opposed to exclusively looking at myoclonus alone [12]. Additionally, most of these studies looked at both outpatient and inpatient censuses, with at least one study to date looking at myoclonus from a population-based perspective [13–14]. A study conducted in 1999 in Olmsted County, Minnesota, found an average annual incidence of 1.3 cases per 100,000 individuals, and that symptomatic myoclonus was more common than essential or epileptic myoclonus, with neurodegenerative diseases being at the forefront of causes of symptomatic myoclonus [13]. Another study conducted in a tertiary referral center in the Netherlands found that more than 40% of individuals presenting with myoclonus were diagnosed with a functional or psychogenic movement disorder after detailed neurophysiologic testing [14].

To our knowledge, very few studies have looked at etiological distributions of myoclonus in an inpatient care setting. Our study seeks to be the first of its kind in providing a detailed review of individuals with myoclonus seen at a large urban inpatient hospital over a 10-year period.

## Methods

### Study Design and Subject Enrollment

The New York Presbyterian Brooklyn Methodist Hospital Institutional Review Committee approved this retrospective cohort study and chart review with a waiver of informed consent due to anonymization of patient data and the impracticality of obtaining informed consent from a large sample (IRB# 1712095). For this retrospective cohort study, patients whose diagnoses included ICD-10-CM codes for “myoclonus” and “asterixis” (G25.3) were compiled on the Electronic Medical Record for New York Presbyterian Brooklyn Methodist Hospital (Brooklyn, New York, United States of America) into an Institutional Review Board approved database that was de-identified for statistical analysis after data collection was completed. Codes for other conditions such as myoclonic epilepsies were not explicitly searched for as these conditions presented primarily in the pediatric population, who were excluded from this study.

### Inclusion/Exclusion Criteria

**Figure 1:**
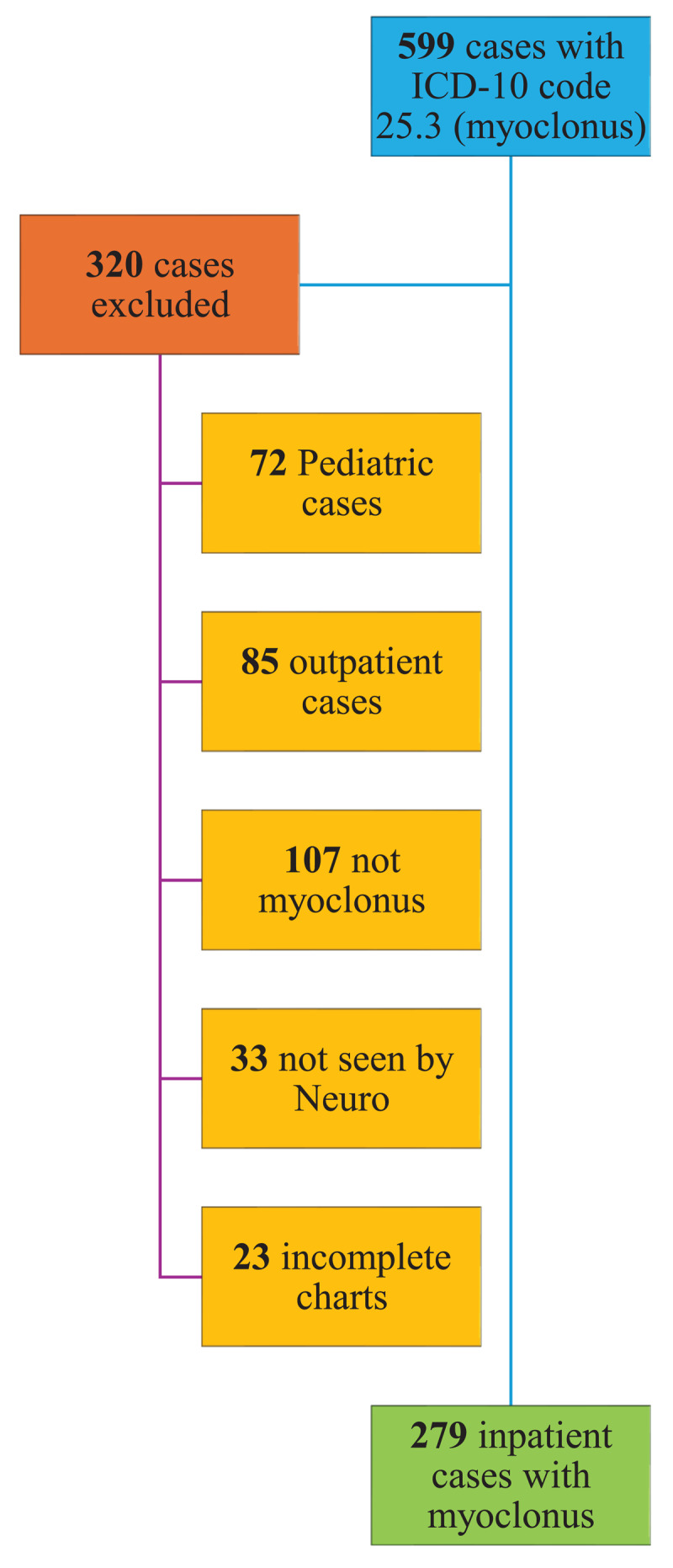
CONSORT (Consolidated Standards of Reporting Trials) flowchart detailing study sample size and exclusion criteria.

Chart data were then screened for inclusion and exclusion criteria, namely adult individuals (defined as individuals 21 years of age or older) seen by a neurologist during their hospital admission from 2010 to 2020 with explicitly documented myoclonus or asterixis in the objective clinical examination and impression/recommendation sections of consultation notes. Pediatric patients (defined as individuals younger than 21 years of age); patients with alternative diagnoses (that is, movement disorders other than frank myoclonus); patients without explicitly documented myoclonus in the charts’ physical examination, assessment, and recommendation sections; patients who were not seen by a neurologist; and patients with limited clinical information were excluded from the study (Figure 1). Although descriptors for myoclonus such as “jerk” or “twitch” were encountered in the history sections of consultation notes, these descriptors were not utilized as inclusion criteria unless the other sections explicitly stated “myoclonus” or “asterixis”.

The records were abstracted for data by five medical residents (D.Z., M.O., A.Z, J.E., and S.R.), a fourth-year medical student (K.O.), and one movement disorder neurologist (M.H.). The movement disorder neurologists (M.H., H.O., H.S., D.V., and M.S.) reviewed all records for standardization of abstracted clinical information and satisfaction of inclusion/exclusion criteria.

### Study Procedures

Included charts were then searched extensively for extractable data, namely the following: demographic data (age at presentation, gender, and ethnicity); duration of symptoms before presentation to the inpatient setting; myoclonus semiology (location and provoking maneuvers); myoclonus etiology (idiopathic, symptomatic, psychogenic, and others); treatment trial and response; and outcome of hospitalization (namely, mortality). Other data collected included associated clinical symptoms (namely gait disturbance, falls, visual disturbances, weakness, sleep dysfunction, cognitive dysfunction, and neuropsychiatric symptoms), past medical history and comorbidities, the presence of polypharmacy (defined in this study as 5 or more concurrent home medications), and examination findings [15].

### Statistical Analysis

Descriptive statistics were summarized as frequency with percentages, and mean, median, range for categorical and continuous factors, respectively. The Chi-square test or Fisher’s exact test was used, as appropriate, to compare the proportions between the variables of interests (gender, ethnicity, race, etiology, myoclonus semiology, etc.) and the outcome (mortality). The relationship between the duration of symptoms or age and other variables were analyzed using Wilcoxon sum rank test. All analyses were performed in SAS Version 9.4 (SAS Institute, Inc., Cary, NC).

## Results

### Demographics

Following stratification for inclusion and exclusion criteria, a total of 279 inpatient individuals – 56.63% female – were included in the study, aging at 70.61 + 15.76 years. Ethnic distribution was 45.52% black, 32.97% Caucasian, and 9.32% Hispanic. While all individuals were diagnosed with myoclonus by a neurologist, 56.3% were not initially diagnosed with myoclonus or a related diagnosis by the admitting medical team. Almost half of the cases studied (49.1%) had 1 ascribable etiology, whereas 36.92% had 2 probable etiologies, 9.68% had 3 etiologies, and less than 3% had 4 or more. A total of 320 charts were excluded from the study: 85 were outpatient cases, 107 had diagnoses apart from frank myoclonus, 72 were pediatric cases, 33 were not seen by a neurologist, and 23 had insufficient chart data available. (Figure 1)

### Etiological Breakdown

**Figure 2:**
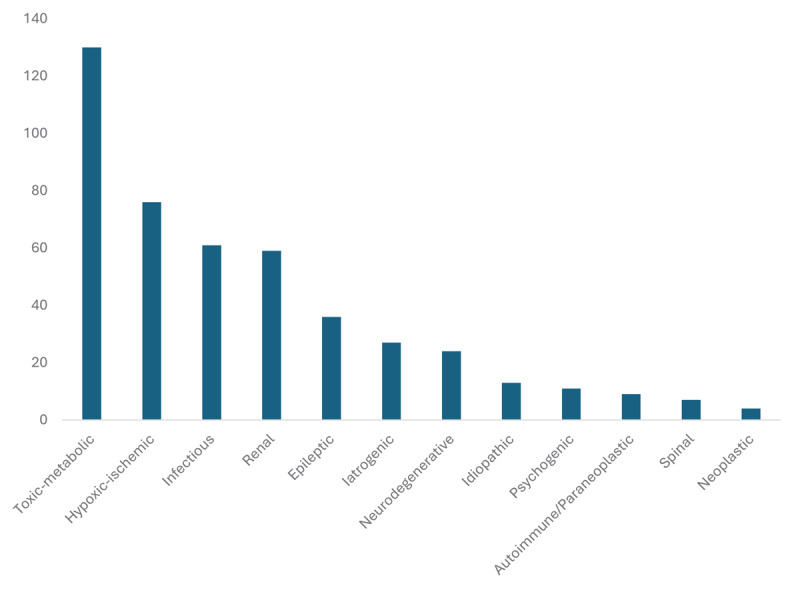
Bar graph detailing number of cases per identified etiology for myoclonus. The total number of charts reviewed was 279 individuals. Almost half of studied cases had one probable etiology, whereas the remainder had 2 or more probable etiologies.

Idiopathic myoclonus accounted for only 4.66% of all cases, whereas psychogenic myoclonus accounted for 3.94% of all cases. The remaining 91.4% of cases were symptomatic, with toxic-metabolic (46.6%), hypoxic-ischemic (27.2%), infectious (21.9%, the majority [27.9%] of which were urinary tract infections and a small fraction [8.2%] were from COVID-19), renal (21.2%), and epileptic (12.9%) etiologies accounting for most cases overall. Less common etiologies for myoclonus in our cohort included iatrogenic (9.68%, with gabapentin use accounting for at least 20% of cases), neurodegenerative (8.60%), autoimmune/paraneoplastic (3.23%), spinal (2.51%), and neoplastic (1.43%) (Figure 2). Toxic-metabolic etiology demonstrated a statistically significant association with gender, with males accounting for more than 50% of cases. Logistical regression analysis yielded older age as strongly associated with toxic-metabolic and infectious etiologies (*p* < 0.05), and younger age being associated more often with autoimmune/paraneoplastic and psychogenic cases. Iatrogenic and renal etiologies were strongly associated with racial background (*p* < 0.05), with the former more common among White and Hispanic individuals and the latter more common among Black individuals. A sample of the final diagnoses per class of etiology encountered is provided in Table 2.

**Table 2:** Sample of final diagnoses encountered per class of etiology.


ETIOLOGY	DIAGNOSES

**Toxic-metabolic**	Hepatic encephalopathy Hyponatremia Hyperglycemic non-ketotic state Diabetic ketoacidosis

**Hypoxic-ischemic**	Anoxic Brain Injury Hypoxic-Ischemic Encephalopathy Lance-Adams Myoclonus Cerebrovascular Disease (Post-Stroke) Intraparenchymal hemorrhage

**Infectious**	Urinary tract infection Septic encephalopathy COVID-19 pneumonia Creutzfeldt-Jakub Disease Cryptococcosis

**Renal**	Uremic encephalopathy Dialysis disequilibrium syndrome

**Epileptic**	Juvenile-onset myoclonic epilepsy Symptomatic myoclonic epilepsy Myoclonic status epilepticus

**Neurodegenerative**	Parkinson’s Disease Multiple System Atrophy Dementia with Lewy Bodies Alzheimer’s Disease

**Iatrogenic**	Gabapentin-induced myoclonus Budesonide/formoterol toxicity Bupropion toxicity Quetiapine toxicity Phenytoin-induced myoclonus Tramadol-induced myoclonus Opiate/narcotic withdrawal Alcohol withdrawal

**Psychogenic**	Psychogenic non-epileptic spells (PNES) Functional myoclonus-like movement disorder

**Autoimmune/Paraneoplastic**	NMDA Encephalitis GAD-65 Antibody-Associated Encephalitis Progressive Encephalopathy w/Rigidity, Myoclonus

**Spinal**	Cervical spinal stenosis Transverse myelitis

**Neoplastic**	Cerebral meningioma Intracranial metastases T-cell lymphoma


### Clinical Characteristics

Of all studied etiologies, autoimmune/paraneoplastic myoclonus, neurodegenerative myoclonus, and spinal myoclonus were associated with a longer duration of symptoms at presentation compared to other etiologies (*p* < 0.05), whereas hypoxic-ischemic etiologies often possessed shorter duration of symptoms at presentation. Toxic-metabolic and renal etiologies had a higher chance of exhibiting a generalized semiology (52.1% and 24.7% of generalized myoclonus cases), whereas focal, multifocal, and segmental myoclonus was more common in epileptic myoclonus cases.

Renal myoclonus was least associated with spontaneity; most cases of renal myoclonus, which were classified as asterixis, were associated with a provoking maneuver. Asterixis was seen more commonly in toxic-metabolic etiologies including renal cases (up to approximately 90% of cases with asterixis), and less often in hypoxic-ischemic and other etiologies. Stimulus-sensitive myoclonus was strongly associated with hypoxic-ischemic etiology (about 65% of cases) and least associated with toxic-metabolic etiology. A total of 48 cases (17% of total) exhibited other coexisting movement disorders such as – in decreasing frequency – tremor, chorea, or dystonia.

### Outcomes and Mortality

Two main outcomes were assessed: symptomatic treatment response and mortality during hospital stay. Treatment response was associated with toxic-metabolic, iatrogenic, and renal etiologies: for example, removal of the offending agent (gabapentin in 20%) in iatrogenic cases was always associated with a good response (i.e. 100% of the time). Hypoxic-ischemic etiology was the most refractory to treatment, be it symptomatic or supportive, with over 60% of individuals not showing any meaningful response to treatment.

In individuals who died during incidental hospital stay for myoclonus, there was a higher chance of hypoxic-ischemic etiology (*p* < 0.001) and a lower chance of neurodegenerative and idiopathic etiologies (*p* < 0.05). About 76% who tried one anti-seizure drug responded to therapy, with lower percentages found for those who tried a second or third anti-seizure drug (48% and 39% respectively). Interestingly, there was an association of higher mortality with non-responders to a third anti-seizure drug (66.7%) compared to those who responded to a second anti-seizure drug (18.2%), with the difference in proportions being statistically significant on Chi-Square testing (*p* < 0.05). Demographic factors (age, gender, and ethnicity), myoclonus duration prior to presentation, myoclonus semiology (location and provoking maneuvers), and anti-epileptic drug use and type had no effect on mortality outcomes (*p* > 0.05).

## Discussion

The primary objective of this retrospective cohort study is to elucidate the distribution of etiologies of myoclonus in a large urban inpatient setting. We found that idiopathic and psychogenic myoclonus were less common than symptomatic myoclonus, with less than 10% accounting for the former two etiologies. Most cases were toxic-metabolic (46.6%), hypoxic-ischemic (27.2%), infectious (21.9%), renal (21.2%), and epileptic (12.9%) in origin. This variation in etiologies mirrors previously cited figures, with symptomatic myoclonus being more common than epileptic and idiopathic etiologies.

This study disclosed novel findings. For example, male individuals predominately presented with cases of toxic-metabolic myoclonus, whereas older individuals exhibited more toxic-metabolic and infectious etiologies of myoclonus. Male preponderance in uremic extrapyramidal syndromes has been reported in previous literature [12]. The divisions of iatrogenic and renal etiologies for myoclonus along racial lines were not reported in prior literature. Additionally, the commonality of renal or uremic myoclonus amongst Black individuals is surprising considering studies citing the absence of racial predilection in uremic encephalopathy with extrapyramidal symptoms [12]. Confounders such as comorbidities (including diabetes mellitus and hypertension), metformin exposure, and thiamine deficiency must also be considered [16].

Semiology of presenting myoclonus correlated with etiology in our study. Whereas toxic-metabolic and renal etiologies exhibited more generalized semiology, likely owing to bilateral or generalized cerebral loci, (multi)focal/segmental semiology was associated with epileptic and vascular/hypoxic-ischemic etiologies, likely owing to focal cortical lesioning. Spontaneity was least associated with renal etiology, as it was responsible for most cases of asterixis (negative myoclonus) observed in this study. Interestingly, about 43.8% of cases of renal-associated myoclonus also exhibited spontaneity, suggesting coexistent pathophysiologic mechanisms described as cortical reflex myoclonus in previous literature [17]. Hypoxic-ischemic injury far more commonly presented itself as stimulus-sensitive compared to other etiologies in our cohort, suggesting brainstem reticular reflex localization [18].

The outcome of individual cases with myoclonus was associated with etiology and the number of anti-seizure drugs trialed. Individuals were more likely to have a non-moribund outcome during hospital stay when exhibiting toxic-metabolic etiologies – as opposed to hypoxic-ischemic etiologies – and response to less than 3 concomitant anti-seizure drugs. Previous studies have demonstrated poor outcomes in individuals exhibiting myoclonus following hypoxic-ischemic cerebral insult [19–20]. The prognosis of status myoclonus associated with hypoxic-ischemic injury is almost universally fatal. However, it must be noted that not all patients exhibiting myoclonus following hypoxic-ischemic injury in our study were in clinical myoclonic status, as approximately 18% did respond to medical treatment.

Despite our study being one of the largest retrospective cohort studies looking at myoclonus in adults in an inpatient setting, it does have some limitations that need to be considered. The first limitation encountered was selection bias. While the Electronic Medical Record’s search engine procured 599 charts, about 320 were excluded, with almost one-third of them not having documented evidence of myoclonus to begin with. Second, the retrospective nature of the study is also a limitation as we had to rely on descriptions made by other neurologists and were not followed by any of us during their hospitalization. Third, the lack of detailed discussion into individual patient diagnoses and patient-specific electrophysiological data recordings of movement disorder semiology limit our certainty regarding the likelihood of the final diagnosis. However, these limitations were ameliorated somewhat with oversight of chart abstraction by neurologists trained in movement disorders. Fourth, 50% of individuals exhibited more than one concurrent explanatory etiology (that is, multifactorial cases made up a substantial proportion of surveyed charts). Drawing conclusions about etiology-specific treatment response can therefore be difficult. Finally, we also had no prospective follow-up from the survivors to assess long-term outcomes of those who did respond to treatment while hospitalized.

In conclusion, we have provided a detailed review of adult-onset myoclonus cases seen over the last 10 years in our large urban inpatient setting, where myoclonus remains a largely underdiagnosed movement disorder. Further research is needed to assess all-cause myoclonus in outpatient and other settings.

## Data Accessibility Statement

De-identified data that support the findings of this study are available from the corresponding author, MH, upon reasonable request.
